# Winter and spring frost events delay leaf‐out, hamper growth and increase mortality in European beech seedlings, with weaker effects of subsequent frosts

**DOI:** 10.1002/ece3.70028

**Published:** 2024-07-21

**Authors:** Lena Muffler, Robert Weigel, Ilka Beil, Christoph Leuschner, Jonas Schmeddes, Juergen Kreyling

**Affiliations:** ^1^ Plant Ecology and Ecosystem Research University of Goettingen Goettingen Germany; ^2^ Ecological‐Botanical Garden University of Bayreuth Bayreuth Germany; ^3^ Experimental Plant Ecology University of Greifswald Greifswald Germany

**Keywords:** acclimation, *Fagus sylvatica*, frost survival, frost tolerance, phenology, provenance trial, seedlings, stress memory

## Abstract

The persistence of plant populations depends crucially on successful regeneration. Yet, little is known about the effects of consecutive winter and spring frost events on the regeneration stage of trees from different seed sources, although this will partly determine the success of climate warming‐driven poleward range shifts. In a common garden experiment with European beech (*Fagus sylvatica*) seedlings from winter 2015/2016 to autumn 2017, we studied how simulated successive spring and winter frost events affect leaf‐out dates, growth performance, and survival rates of 1‐ to 2‐year‐old seedlings from provenances differing in climate at origin. We further investigated the combined effects of successive frost events. The first spring frost after germination led to a mortality rate up to 75%, resulting in reduced seedling numbers but better frost tolerance of the survivors, as reflected in a weaker impact of the following winter frost event in the survivors compared to the non‐acclimated control. Final plant height was most strongly reduced by the spring frost in the second year. The winter frost event delayed leaf‐out by up to 40 days, leading to severe growth impairment in 2017. Our results indicate partly successful frost acclimation and/or the selection of frost‐hardier individuals, because the negative growth effects of consecutive frost events did not add up after exposure to more than one event. Both mechanisms may help to increase the frost tolerance of beech offspring. Nevertheless, mortality after the first spring frost was high, and frost exposure generally caused growth reductions. Thus, achieving higher frost tolerance may not be sufficient for beech seedlings to overcome frost‐induced reductions in competitive strength caused by winter frost damage and delayed leaf enfolding.

## Graphical abstract

Despite global warming, frost events may still occur, which could hamper warming‐driven northward range shifts of tree species. Our study showed that frost may act as an environmental filter in the seedlings' first year contributing to an increased frost tolerance in beech offspring, but mortality was still high and growth reduced due to frost events. Thus, increased frost tolerance may not be sufficient to overcome detrimental effects on the competitiveness of beech, when regenerating beyond its current cold distribution margin, and therefore may indeed limit a northward range expansion.

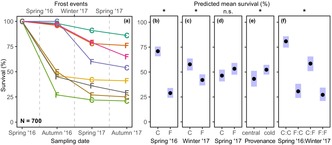

## 1 INTRODUCTION

The growth of temperate tree species is limited by various climate factors across their distribution ranges. High temperatures and drought events typically hamper tree growth at the equatorial edge of the species' distribution ranges, leading to population decline upon climate warming (IPCC, 2018; van der Maaten et al., 2017). Growth in cold habitats, on the other hand, is assumed to be limited by cold events (Sakai & Weiser, 1973), which may continue to happen with the same intensity and frequency in the future, despite global warming (Kodra et al., 2011; Petoukhov & Semenov, 2010). However, if and how cold events limit tree growth, and the related mechanisms, are controversially discussed. Some authors assume minimum temperatures as the limiting factor and that the cold tolerance is higher in tree species of colder origin (Kreyling et al., 2015; Sakai & Weiser, 1973). With respect to likely continuing growth limitation by cold temperatures, other studies suggest the spring frost risk during and shortly after budburst as the critical factor, which could prevent growing season extension and thus increased productivity in a warming climate (Kollas et al., 2014; Lenz et al., 2013; Muffler et al., 2016; Vitasse et al., 2014). In view of the growth‐limiting influence of potentially persisting cold events, predictions of tree growth performance and of how rapidly tree species will migrate beyond their current cold distribution margin during climate change should thoroughly consider cold‐season temperature scenarios (Lenoir et al., 2008; Parmesan, 2006; Sykes et al., 1996).

In general, extreme climate events such as severe drought and frost have a greater impact on plant fitness than gradual climatic changes, to which plants can acclimate and adapt to a certain degree (Walter et al., 2013). Consequently, more precise predictions of the future response of tree species to climate change are only possible, if the response to such extreme events, in particular to highly damaging extreme frost events, is understood (Studd et al., 2021). Climate warming leads to an increasing frequency of extreme thermal events such as sudden and very pronounced swings in winter temperature (‘winter weather whiplashes’; Francis et al., 2022) and successive frost events, which meets a growing plant vulnerability to spring frost damage, as leaf‐out happens earlier (Ma et al., 2019; Zohner et al., 2020). Consequently, studies examining the response of trees to successive extreme frost events are urgently needed.

Theoretically, successive extreme climate events can impact plant physiology in two different ways, either by reducing stress tolerance through legacy or carry‐over effects due to reversible or irreversible impairment of biochemical processes that lead to increased damage during a subsequent extreme climate event, or by increasing stress tolerance in a following event through acclimation (Walter et al., 2013). This acclimation to a subsequent event of the same kind may happen for example through epigenetic genome modifications (Bruce et al., 2007; Chinnusamy et al., 2008; Goh et al., 2003). Ambiguous results have been obtained with respect to the effect of frost stress, which is generally less studied. For example, Knight, Trewavas & Knight (1996) found evidence for frost acclimation, while e.g. Polle et al. (1996) reported reduced tolerance to subsequent frost events. These contrasting results may be due to the fact that plant species seem to differ in their ability to develop a so‐called stress memory for frost, which could lead to acclimation (Kong & Henry, 2020). So far, a clear picture has not emerged due to the low number of studies conducted on the effects of multiple successive frost events on trees.

Tree species persistence and the establishment of new populations crucially depend on the capability for successful regeneration. Young plants may respond more sensitively to climate extremes than adults because they have smaller root systems or store less carbohydrates, as, for example, is indicated by a higher frost sensitivity of leaf tissues of beech seedlings compared to adult trees (Hofmann et al., 2014). Given a higher sensitivity at juvenile age, strict environmental filtering by a harsh environment will exert a strong selection effect during the establishment process of tree offspring by favouring the best performing individuals (Petit & Hampe, 2006). Thus, such a filtering will greatly reduce the number of surviving young trees, but it would produce a population with individuals more tolerant to the stressor (Petit & Hampe, 2006).

The effect of cold temperatures on plant performance and plant fitness is largely driven by the timing of frost events and plant phenology (Chuine & Beaubien, 2001; Körner et al., 2016; Kramer et al., 2000; Muffler et al., 2016). With respect to leaf phenology, two mechanisms are responsible for triggering leaf‐out in tree species. Besides the influence of photoperiod, the leaf‐out date of tree species depends on the accumulation of periods with cold temperatures in the autumn and winter (chilling) and the accumulated time with warm temperatures in the spring (forcing) (Baumgarten et al., 2021; Körner & Basler, 2010). With increasing average temperatures due to climate warming, the start of the growing season and spring leaf‐out of plants are advancing (Liu et al., 2022; Menzel, 2000; Menzel & Fabian, 1999). Many studies have focused on the effects of increasing temperatures on the leaf‐out of tree species (e.g. Fu et al., 2018; Geng et al., 2020). However, short but extreme cold snaps in winter may persist into the future or even increase in intensity in northern temperate continental areas, despite or even because of climate warming (Francis et al., 2022; Kodra et al., 2011). The effect of such cold snaps in winter on spring leaf‐out is not well covered by phenological studies.

European beech (*Fagus sylvatica* L., hereafter termed beech) is the dominant native tree species in Central Europe, growing under a wide range of environmental conditions (Bolte et al., 2007; Leuschner et al., 2006). In many Central European countries, beech is one of the most common tree species, which is of considerable economic importance. The distribution range of beech is projected to shift northwards due to increasing temperatures and increasing atmospheric evaporative demand with climate change (van der Maaten et al., 2017). However, besides drought, beech is also sensitive to frost. The local adaptation potential to cold events is controversially discussed, however. Višnjić and Dohrenbusch (2004) found in a common garden experiment local adaptation to frost with respect to the freezing tolerance of buds, and Kreyling et al. (2014) confirmed this with respect to winter frost survival. In apparent contradiction, Malyshev et al. (2018) did not observe significant adaptation of the beech individuals of cold‐marginal populations in terms of winter dormancy depth and budburst forcing requirements. In comparison to the local adaptation of southern beech provenances to drought (e.g. Rose et al., 2009; Thiel et al., 2014), local adaptations to cold events in provenances at the cold distribution edge are not yet so well studied. Moreover, it is still under debate, which kind of frost events affects beech most. Several studies found a high frost sensitivity of freshly sprouted leaves in spring (Körner et al., 2016), leading to spring frost‐induced growth reduction of up to 50% and a growth recovery only in the subsequent year (Dittmar et al., 2006; Príncipe et al., 2017), whereas mid‐winter frost was regarded as less important for leaf buds due to their high frost tolerance in winter (Lenz et al., 2016). In contrast, beech stem growth of mature trees at the north‐eastern distribution edge was found to respond sensitively to severe winter frost (Augustaitis et al., 2015; Matisons et al., 2017; Weigel et al., 2018), and the distribution range of beech correlates with winter cold there, e.g. a mean January temperature > −3°C is required for the species' survival (Bolte et al., 2007). Here, frost might act indirectly through fine root damage and reduced root resource uptake activity (Reinmann et al., 2019; Sanders‐DeMott et al., 2018), rather than directly through damaged buds or cambial meristems, given the high frost tolerance of these tissues in winter (Lenz et al., 2016). However, only few studies on the impact of frost events on beech growth at the cold distribution edge do exist so far (e.g. Weigel et al., 2018, 2021), in particular at the regeneration stage.

The lack of profound knowledge about the impact of winter and spring frost on beech regeneration and growth is critical in view of both the predicted climate warming‐driven range expansion of beech beyond its current cold distribution edge and the earlier onset of leaf emergence due to increasing temperatures as discussed above. To fill this gap, we conducted a common garden experiment to study the impact of three consecutive frost events on central and cold‐marginal beech populations in their first 2 years after germination. We aimed at quantifying the isolated effects of winter and spring frost events and their interaction on spring phenology, growth performance and survival, and explored effects of successive frost events. We hypothesized that (1) winter and spring frost events negatively affect fitness traits in beech, such as delaying phenology, limiting growth performance, and reducing survival; (2) frost exposure reduces survival rates more in the first than in the second year; (3) the first and/or second frost event result in a higher frost tolerance during the subsequent second and/or third frost event; and (4) seedling mortality, growth reduction, leaf necrosis, and the delay of leaf‐out dates due to frost will be greater in central than in cold‐marginal populations.

## 2 MATERIALS AND METHODS

### 2.1 Experimental setup and provenance origin

**FIGURE 1:**
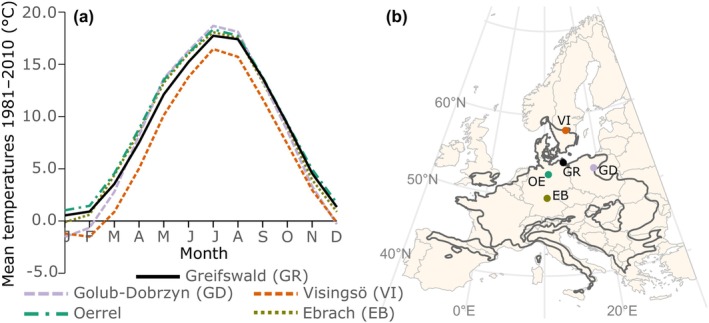
(a) Monthly mean temperatures (Haylock et al., 2008) for the experiment location (Greifswald) and the origin of the four beech provenances; (b) Location of the experiment (black dot) and provenance origins within the closed distribution range (dark grey outline) of European beech (according to: Caudullo et al., 2017).

The common garden study was conducted in the open‐air experimental garden next to the Botanical Institute in Greifswald, north‐eastern Germany (54.10° N, 13.36° E), between winter 2015/2016 and autumn 2017. We used beech nuts from the distribution centre (Oerrel, north‐west Germany, and Ebrach, southern Germany), where annual and cold‐season temperatures are similar to the conditions in Greifswald (Figure 1a,b; Table 1). For comparison, beech nuts from populations from the eastern and northern cold distribution edge (Golub‐Dobrzyn, northern‐central Poland, and Visingsö, southern Sweden) were cultivated. Visingsö is colder than the central provenances in all seasons, whereas Golub‐Dobrzyn is colder especially in the winter months (Figure 1a,b; Table 1). The seeds were collected in Germany in autumn 2014, in Poland and Sweden in autumn 2015, depending on local seed production. All seeds were collected in natural beech forests and stored in dry state in closed plastic bags in the refrigerator. For germination, the seeds were inserted in seed trays (50 × 32 × 6 cm; divided into six sections, one provenance per section, 10 beech nuts per section) filled with moist, slightly silty sand substrate (pH = 7.1 and C/*N* = 16) in winter 2015/2016. The seeds were covered with a fleece to simulate natural growing conditions under leaf litter and to protect the seeds from frost. After *N* = 700 seeds had germinated, the seedlings were transplanted individually into 0.70 L‐pots (7 × 7 × 18 cm) filled with the same substrate. It is noteworthy that the seed material collected in 2015 germinated 2.7 times as good as the seeds collected in 2014. It is unclear whether this is due to different seed qualities in the 2 years, or was caused by different periods of storage. This difference had no effect on our experiment, as we worked with the final sample of *N* = 700 potted seedlings out of the total number of initial beechnuts (originally *N* = 3200 in total; 800 per provenance origin). During the subsequent winters, the pots with the seedlings were buried in sandy substrate to simulate natural soil temperature conditions and to protect the roots from harsh soil frost. Further, natural shading conditions were simulated by reducing sunlight to approximately 45% of open‐sky conditions, using a net in the fully sun‐exposed experimental garden. For the experiment, the pots were placed on level terrain in an area of 50 m^2^ in sufficient distance to taller trees and buildings to avoid the influence of lateral heat sources and guarantee similar thermal conditions for all pots (see Figure S3 for temperature measurements from two different corners of the experimental garden). When it was not raining, we regularly checked that the soil in the pots did not desiccate, relying on horticultural expertise. If needed, the plants were watered manually with a hose equipped with a nozzle to prevent drought stress, which guaranteed that soil water availability did not influence frost hardiness in the experiment.

**TABLE 1:** Comparison of climate conditions at the experimental site (common garden) and at the origin of seed provenances (30‐year averages, 1981–2010) (Haylock et al., 2008).

	Mean annual temperature (°C)	Annual precipitation sum (mm)	Mean February temperature (°C)
Greifswald (common garden)	8.7	577	0.9
Oerrel (provenance)	9.3	679	1.5
Ebrach (provenance)	8.8	671	0.6
Golub‐Dobrzyn (provenance)	8.4	510	−0.6
Visingsö (provenance)	6.8	547	−1.5

### 2.2 Frost scenarios

**FIGURE 2:**
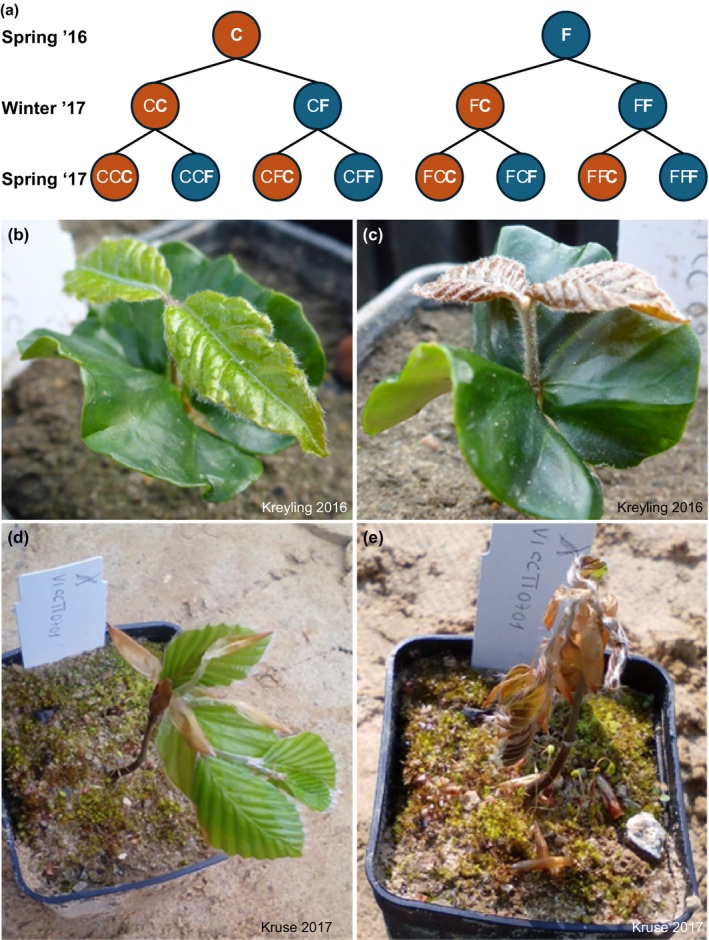
(a) European beech seedlings were kept under ambient conditions as control (C) or exposed to frost (F) during three artificial frost events in spring 2016 (May–June), winter 2017 (February), and spring 2017 (May–July), with division of each group into (C and F) for each event. Seedlings right before (b, d) and after (c, e) the spring frost treatment in 2016 (b, c) and in 2017 (d, e).

We simulated one winter and two spring frost events to analyse the individual and potentially interacting effects of these events on plant performance (see Figure 2 for a diagram of the timeline and combination of frost treatments). To do so, we exposed the plants to either a single winter or spring frost event or no frost event, or a combination of these stressors. To explore how winter or spring frost affects frost tolerance in a subsequent spring frost event, we applied spring frost in two consecutive years. In this design, both stress combinations (winter frost followed by spring frost and spring frost followed by spring frost) were alternatively applied. In another treatment, we applied all three frost events to the plants. The experiment hence consisted of eight factorial combinations of frost or control conditions (no frost) over two spring and one winter treatment period in a fully‐crossed experimental design (treatments FFF, FFC, FCF, FCC, CFF, CFC, CCF, CCC with frost: F, control: C during the three‐time intervals spring 2016‐winter 2017‐spring 2017; Figure 2). The potted seedlings were exposed to frost events of one‐night duration in climate chambers (LT‐36VLX, Percival, Perry, Iowa, USA), which took place in the following time intervals: spring frost in 2016 (17 May–29 June at −4.6 ± 1.1°C), winter frost in February 2017 (25–26 February at −13.3 ± 1.7°C; referred to as the *Winter* ‘17 treatment because it was carried out in February ‘17), and spring frost in 2017 (3 May–13 July at −4.3 ± 0.6). To protect the roots from winter frost, the pots were insulated with Styrofoam (polystyrene foam). The spring frost exposure was applied individually to each plant directly after unfolding of leaves according to the BBCH Code 15 (Hack et al., 1992), which is why the last sub‐sample of plants was subjected to the frost treatment in June and July, as described above. Our research question made it necessary to wait in all experimental plants long enough to test the influence of late frost precisely at the point of time when leaves emerged, instead of measuring the frost effect at an arbitrary point of time, when frosts usually happen in May and early June, without paying attention to the phenological development stage. Thus, we assessed the phenological stages of the plants continuously (three times per week), and the date of frost application was assigned to each plant individually according to its leaf‐out date, regardless of the treatment group it belonged to. In the climate chambers, the temperature was gradually lowered at a cooling rate of 1.8 K/h for the spring frost treatment and at 2.3 K/h for the winter frost treatment until the final minimum temperature was reached. We chose a minimum spring temperature of −4°C to −5°C, which is within the lower temperature range that young leaves of European beech have been found to tolerate during and directly after budburst (Lenz et al., 2013; Vitra et al., 2017). A value of about −13°C was chosen for the winter temperature, because this level represents realistic frost scenarios for central to cold‐marginal provenances, while being more negative than the usual average temperature conditions during this time of the year (Figure S1). Further, this temperature level was selected to maintain sublethal conditions above the cold tolerance of beech (−20°C or less) during this time of the year (Kreyling et al., 2015). Sublethal effects have recently been shown to be of ecological importance (Malyshev & Henry, 2012) and may be biologically more influential than lethal effects observed in unrealistically harsh frost experiments. The chosen minimum temperature was maintained for 2 h during the spring frost treatment and for 1 h during the winter frost treatment, after which the plants were returned to ambient temperature, applying a warming rate of 4.3 K/h in the spring frost treatment and of 3.8 K/h in the winter frost treatment. When returned to the experimental garden, the plants were again distributed in a randomized block design. The simulated frost magnitude exceeded the ambient frost conditions by at least 2°C in all cases and on average by around 11°C during the time of treatment application and during the 30 days prior to it (Figures S2 and S3) as measured with TRIX‐8 thermistor–data logger units (LogTag, Lafayette, New Jersey, USA). We additionally measured the temperature in the climate chambers with the same TRIX‐8 thermistor–data logger units.

### 2.3 Response variables

We visually assessed individual seedling survival (dead or alive) between the frost simulations, i.e. in autumn 2016, in spring 2017 between the winter and the spring frost simulation, and finally in autumn 2017. We verified that trees classified as dead did not resprout later. Further, we measured seedling height with a tape measure along the stem axis from the base of the stem to the highest living bud late in September 2016 and 2017. We also calculated height increment rates in 2017 by comparing the seedling height in 2017 and in 2016 (height measured in 2017 minus height measured in 2016). Negative increment in 2017 was assigned to tip necrosis. We regularly observed the leaf phenology three times a week in 2016 and 2017 from May to July. In 2016, spring leaf‐out was determined for each individual when the cotyledons and the first pair of leaves were fully unfolded, and in 2017, when five leaves were completely unfolded according to the phenological scale BBCH 15 (Hack et al., 1992) (see also Figure 2 for examples).

### 2.4 Statistical analysis

We applied a 4‐factorial analysis of variance (ANOVA) to isolate the main effects of each of the three separate frost treatments plus the provenance effect and of all 2‐fold *treatment*: *treatment* and *treatment*: *provenance* interactions on each response variable (survival in 2016 and 2017, height in autumn 2017, necrosis, spring leaf‐out dates). The provenances were categorized to the groups “central” (EB, OE) and “cold” (GD, VI) in all models to achieve robust sample sizes in the sub‐groups. Model comparisons showed that this categorization in two provenance groups instead of four separate provenances yielded better model fits for all response variables (model comparison with Akaike's Information Criterion; Akaike, 1974). If we found significant (*F*‐test with *p* < .05) interaction effects of the treatment factors, we computed the estimated marginal means (EMMs) for each factor combination (R package emmeans v1.7.4; Lenth, 2022) to assess, which factor combinations differed significantly from each other at *p* < .05 (indicated by "*" in graphs). For graphical comparison of the interactions, EMMs are shown together with their predicted 95% confidence interval around the predicted marginal mean. The residuals of each ANOVA were tested for normal distribution with the Shapiro–Wilk‐test, Q–Q plots, and histograms. Due to non‐normally distributed residuals in case of leaf‐out dates, we applied a Kruskal–Wallis rank sum test of single factors instead of a standard ANOVA. Homogeneity of variances was assessed with Levene's test (R package DHARMa v0.4.5; Hartig, 2022). Due to non‐homogeneity of variances in case of height meeting mortality‐related uneven sample sizes across treatments, we stratified the data to achieve equal sample sizes. This means that we applied random sampling to reduce the sample size in each of the 8 possible treatment combinations to *n* = 23, which was the sample size in the smallest treatment combination. For the individual binomial survival probability (0: dead and 1: alive) and for the individual probability to develop necrotic shoot tips (0: no necrosis and 1: necrotic tip), we fitted generalized linear models with a logit link function and Chi‐squared significance test in the ANOVA. Overdispersion was not detected for these models in a simulation‐based dispersion test (DHARMa). For the graphical representation of the data variance, we calculated the standard deviation of the mean for the number of individuals with necrotic tips in a 1000‐fold bootstrapping procedure. The regression of increment and spring leaf‐out dates was assessed with Spearman's rank correlation coefficient. All analyses were performed in R v4.04 (R Core Team, 2021, Vienna, Austria).

## 3 RESULTS

**FIGURE 3:**
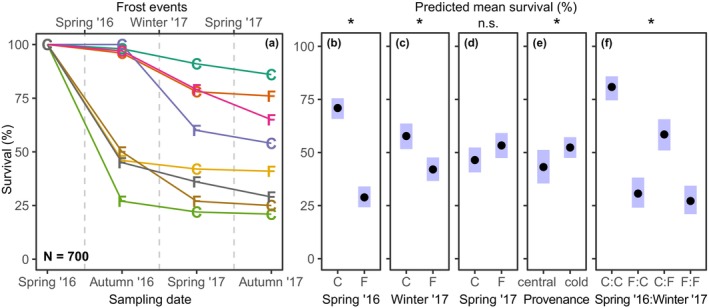
(a) Percentage of surviving seedlings compared to the start point (=100%) in the eight frost treatments during the investigation period (spring 2016–autumn 2017). The type of treatment (C: control, F: frost treatment) refers to the frost event preceding a sampling date. The date of the three frost simulations is indicated on the top y‐axis and the interjacent sampling dates are on the bottom y‐axis; (b–f) Predicted mean survival rate (with 95% confidence intervals) for all main effects and the significant interaction. The main effects in (b–e) were isolated by ANOVA.

Survival probability decreased by 42% (control minus treatment; Figure 3b) in response to the first spring frost exposure in 2016, which is considerably more than due to any of the subsequent frost events (Figure 3a–d; Table 2). While the frost exposure in winter 2017 still had a weak but significant effect on survival (16% decrease in the frost treatment compared to control and non‐overlapping confidence intervals of the predicted group means), the last frost event in spring 2017 did not reduce survival further (Figure 3c,d; Table 2). Overall, the cold provenances showed a 9% higher survival rate than the central provenances, although the group means were only marginally different from each other (overlapping confidence intervals of the predicted group means; Figure 3e). If seedlings were exposed to frost in spring 2016, this frost impact was so influential that no effect of the subsequent, interacting frost exposure in winter 2017 was visible anymore (Figure 3f). Frost in winter 2017 had a stronger effect on those seedling's survival that had not faced any frost before than on seedlings that had already experienced and survived late frost in spring 2016.

**TABLE 2:** Analysis of variance and deviance tables for survival probability, plant height in autumn 2017 (*F*‐test) and the binomial survival and necrosis probabilities (Chi‐squared test) in dependence on all individual treatment factors and all their two‐fold interactions.

Response	Explanatory	df	Deviance	Resid. df	Resid. Dev	*p* Value
Survival probability	Null model			699	970.38	
	**Spring ‘16**	**1**	**122.158**	**698**	**848.22**	**.001**
	**Winter ‘17**	**1**	**14.2**	**697**	**834.02**	**.001**
	Spring ‘17	1	3.665	696	830.36	.056
	**Provenance**	**1**	**3.957**	**695**	**826.4**	**.047**
	Spring ‘16:Spring ‘17	1	2.088	694	824.32	.15
	**Spring ‘16:Winter ‘17**	**1**	**7.308**	**693**	**817.01**	**.0069**
	Winter ‘17:Spring ‘17	1	0.318	692	816.69	.57
	Spring ‘16:provenance	1	0.119	691	816.57	.73
	Spring ‘17:provenance	1	0.308	690	816.26	.58
	Winter ‘17:provenance	1	0	689	816.26	.99

			Sum Sq	Mean Sq	*F* value	
Height	**Spring ‘16**	**1**	**555.8**	**555.8**	**19.3**	**.001**
	**Winter ‘17**	**1**	**240.6**	**240.6**	**8.3**	**.0044**
	**Spring ‘17**	**1**	**1578.9**	**1578.9**	**54.8**	**.001**
	Provenance	1	67.5	67.5	2.3	.13
	Spring ‘16:Spring ‘17	1	5.8	5.8	0.2	.66
	Spring ‘16:Winter ‘17	1	106.5	106.5	3.7	.056
	**Winter ‘17:Spring ‘17**	**1**	**237.1**	**237.1**	**8.2**	**.0046**
	Spring ‘16:provenance	1	1.3	1.3	0.0	.83
	Winter ‘17:provenance	1	5.4	5.4	0.2	.67
	Spring ‘17:provenance	1	38.5	38.5	1.3	.25
	Residuals	173	4986.4	28.8		

			Deviance	Resid. df	Resid. Dev	
Necrosis probability	Null model			370	350.4	
	Spring ‘16	1	2.0	369	348.4	.15
	Winter ‘17	1	0.9	368	347.5	.34
	**Spring ‘17**	**1**	**43.9**	**367**	**303.6**	**.001**
	**Provenance**	**3**	**4.0**	**366**	**299.6**	**.045**
	Spring ‘16:Spring ‘17	1	2.2	363	297.1	.14
	Spring ‘16:Winter ‘17	1	0.1	362	297.0	.70
	Winter ‘17:Spring ‘17	1	2.9	361	294.1	.089
	Spring ‘16:provenance	3	1.8	358	292.3	.61
	Spring ‘17:provenance	3	4.9	355	287.4	.18
	Winter ‘17:provenance	3	0.5	352	286.8	.91
					χ^2^	
Leaf‐out date 2017	Spring ‘16	1			**6.3**	**.01**
	Winter ‘17	1			**67.4**	**.001**

*Note*: The full model output is given for each response variable separately. Significant terms (*p* Value < .05) in bold.

Abbreviations: df, degree of freedom; Mean Sq, mean sum of squares (sum Sq/df); Resid. Dev, residual deviance; Resid. df, residual degree of freedom; Sum Sq, sum of squares.

**FIGURE 4:**
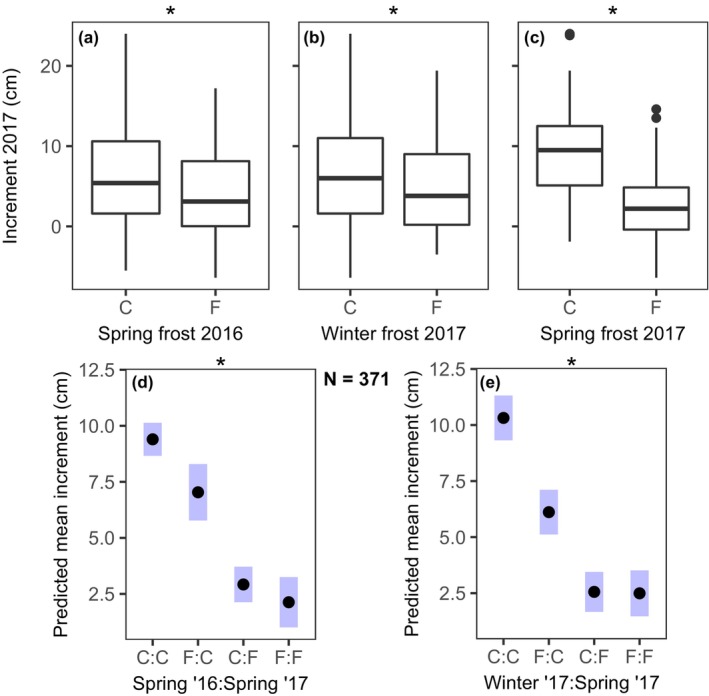
(a–c) Plant height growth (increment, cm) during 2017, which is the increment from autumn 2016 to autumn 2017, in dependence on the significant main treatment effect (a–c; C, control vs. F, frost treatment) and (d, e) Predicted mean increment (cm) (with 95% confidence intervals) for the significant interaction of winter ‘17× spring ‘17 frost events. The main effects in a–c were isolated by ANOVA. Negative growth rates result from shoot necrosis (Figure 5).

All types of frost events significantly decreased the final increment of the seedlings across provenances in 2017 (Figure 4a–c; Table 2). The late‐frost exposure in spring 2017 had the strongest main effect, decreasing the increment to around a quarter of the control on average (Figure 4c; Table 2), and final height in autumn 2017 was reduced by around 40% due to the frost effect (Figure S4). Regarding the interaction between two subsequent frost events, the impact of the spring 2017 frost event was so strong that no additional effect of the previous spring (2016) or winter (2017) frost exposure could be seen in the group that experienced the spring frost in 2017 (F:F and C:F vs. F:C in Figure 4d,e). For example, in the interaction of winter and spring frost, the effect size of the spring frost 2017 halved in the individuals that had experienced the preceding 2017 winter frost, compared to the individuals in the control group (Figure 4e). Comparing the final plant height in autumn 2017 yielded similar results, with the frost exposure in spring 2017 leading to the smallest individuals at the time of harvest (Figure S4). Notably, no additional effect of the previous frost events in spring and winter could be seen in the group that experienced spring frost in 2017, i.e. the single negative effects of each separate frost event on growth did not add up in this group, as visible in the interaction effects (Figure 4d,e).

**FIGURE 5:**
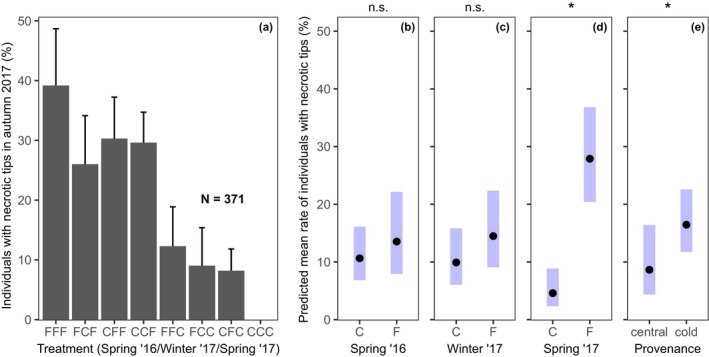
(a) Percentage of individuals with necrotic shoot tips in autumn 2017 (negative increment in 2017) in the eight treatments (C: control vs. F: frost treatment in the sequence spring 2016–winter 2017–spring 2017; for example, “FFF” stands for individuals exposed to all three frost treatments, “CCC” for no exposition to any frost event, and “FCC” for frost exposure only in spring 2016, but not afterwards). Given are the mean (bar) and standard deviation of the data according to a 1000‐fold bootstrap procedure. (b–e) Predicted mean proportion of individuals with necrotic tips (in %) and their predicted 95% confidence intervals around the mean for the main treatment effects. The main effects in (b–e) were isolated by ANOVA.

Individuals that were not exposed to any frost treatment showed no necrotic shoot tips, while almost 40% of the individuals with exposure to all three frost treatments were affected by necrotic tips in autumn 2017 (Figure 5a). Again, the largest increase in necrotic shoot tips happened after the 2017 spring frost event (Figure 5d), whereas the earlier frost events themselves had no effect and were associated with a much lower proportion of necrosis (Figure 5b,c; Table 2). The cold provenances showed a slightly elevated necrosis probability compared to the central provenances, although this effect was not much stronger than the effect of the non‐significant frost events (overlapping confidence intervals of the predicted group means; Figure 5e).

**FIGURE 6:**
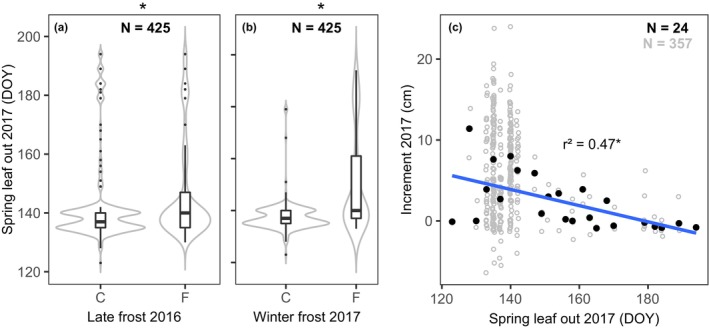
(a, b) Individual spring leaf‐out dates during 2017 in dependence on the frost simulation (C: control vs. F: frost treatment) in spring 2016 and winter 2017. The frost event in spring 2017 is not included here, as it was applied after leaf out. (c) Relationship between spring leaf‐out and increment rate during 2017 in the sample of seedlings (grey dots: all individual relationships, black dots: values averaged for each spring leaf‐out date over which the regression (blue line) was calculated). The main effects in (a, b) were isolated by ANOVA.

Both the frost treatment in spring 2016 (Table 2) and in winter 2017 (Table 2) delayed leaf‐out in 2017 by about 3 days (median value, Figure 6a,b). Despite the seemingly small response to the frost exposure on average, the frost exposure in winter 2017 shifted leaf‐out of the latest‐developing individuals far into the growing season (Figure 6a,b), with 25% of the individuals in the control group unfolding leaves after May 20, while the same proportion of frost‐exposed individuals had leaf‐out at least 21 days later (after June 10). The corresponding difference in leaf‐out dates for the latest 10% of individuals in each group was over 40 days. This pronounced delay in leaf development also affected the growth performance, because height increment rates in 2017 decreased strongly, from about 5 cm annual increment in the early‐flushing individuals to zero increment with later leaf‐out dates in and after July (Figure 6c).

## 4 DISCUSSION

In support of our first hypothesis, we found that the first frost event in the spring after germination and leaf‐out more than halved the plants' survival. Similarly, the more frost‐hardy seedlings survived, as is reflected in the reduced impact of the following winter frost event on these survivors (Figure 3). The lacking effect of the second spring frost on survival is in line with our second hypothesis, because the impact on survival was higher in the first year than in the second year (Figure 3). In addition, as postulated in our third hypothesis, the negative effects of each separate frost event did not add up, when the plants experienced several frost events. In other words, the treatment that experienced the last plus any of the previous frost events showed the same growth reductions as the treatment that experienced only the last (Figure 4 and Figure S4). Most unexpected was the strong effect of the single‐night winter frost on the phenological timing of leaf‐out. For some individuals that experienced winter frost in 2017, leaf‐out was delayed by up to 40 days (Figure 6). This delayed leaf‐out, together with the smaller effect of the spring frost 2017 in the group that had experienced winter frost before, may hint at acclimation to the simulated successive frost events through dormancy deepening to increase frost hardiness (North & Kovaleski, 2024). However, the tremendous delay in leaf‐out resulted in almost no height increment in 2017 due to the shorter growing season length, as was similarly observed by Baumgarten et al. (2023), who manipulated leaf‐out by applying different multi‐day frost scenarios. In contradiction to our fourth hypothesis, our results provide only little evidence for a better frost adaptation of the cold‐marginal provenances compared to the central ones (lacking interaction term of frost treatments with the factor *provenance*; Table 2). Overall, our study provides new insights into the potential of juvenile beech trees to acclimate to successive frost events. The results suggests that frost exposure during the critical stages of early leaf emergence and delayed leaf emergence due to winter frost exposure hamper the regeneration process and reduce the competitiveness across seed sources.

### 4.1 Effects of winter and spring frost events on the survival and growth performance of beech offspring

The spring frost event with minimum temperatures of around −4.6°C shortly after leaf‐out resulted in a high mortality of up to 75% in first‐year seedlings in 2016 (Figure 3). Moreover, the survival rate of the individuals that did not experience spring frost in 2016 but were exposed to winter frost (around −13.3°C) in February 2017, also declined by around a third compared to the control, while the effect of this winter frost was much smaller, when individuals had experienced the previous spring frost in 2016 (Figure 3). These results suggest that the spring frost in the first year might have had a selective effect on the seedlings, so that the more frost‐tolerant individuals survived the subsequent winter frost event. One possible explanation is that frost events in the first year increased the share of more frost‐resistant individuals in the remaining population due to a strong selection pressure on juvenile beech trees, whereby the best performing individuals survived (Petit & Hampe, 2006).

Frost is generally a strong environmental filter for germination and seedling emergence (Muffler et al., 2021). The presence of local adaptation to frost in provenances from cold environments is supported by studies on beech winter frost survival of Kreyling et al. (2014), whereas Malyshev et al. (2018) found that individuals of cold marginal provenances show no adaptation with respect to winter dormancy and budburst forcing. Yet, we did not find a significant interaction between population origin and the type of frost treatment in our comparison of central and cold‐marginal populations (Table 2), which may question the significance of frost adaptation at least in our beech provenances. In turn, in light of the lacking provenance effect on spring phenology, we also found no evidence of maladaptation of colder provenances to warming, which could be at higher risk of spring frost damage in future due to genetically determined earlier leaf emergence compared to warmer provenances, as was found for other tree species (Mura et al., 2022).

The observed sensitivity of seedlings to frost events, visible in the low survival rate during the first year, questions the assumed potential of beech to establish beyond its current northern distribution limit. Nevertheless, our results from seedlings and the specific spring frost conditions in the experiment may not be simply transferred to mature trees. Still, field studies have shown that spring frost can severely impair the vitality and growth performance of mature beeches, causing dieback of twigs and branches and growth reductions of up to 50% (Dittmar et al., 2006; Príncipe et al., 2017). Our results suggest that the thermal niche of juvenile plants is even narrower than that of the adult trees, shown by the stronger frost sensitivity (Hofmann et al., 2014; Jackson et al., 2009). This may be attributed to the relatively small leaf area and root biomass of seedlings and thus lower carbohydrate supply, which could limit the capacity for damage repair, and the position near the ground, where frost is usually more severe than for tissue at greater height (D'Andrea et al., 2019; Marquis et al., 2020). How important the frost‐induced loss of foliage is for seedling vitality, is demonstrated by the associated drastic increase in mortality, as shown in Figures 2 and 3. In contrast, two‐year‐old seedlings apparently were more resilient to damaging frost events than one‐year‐old ones, possibly due to a higher photosynthetic capacity and assumed larger carbohydrate reserves. This might explain, why the second spring frost event in 2017 did not negatively impact the survival of the two‐year‐old seedlings (Figure 3). In any case, although the 2‐year‐old seedlings showed a higher survival rate than the 1‐year‐old plants, their growth performance was still notably affected by frost exposure (Figure 4). These observations highlight that the impact of frost on the vitality and survival of juvenile trees is highly dependent on the development stage. Our study offers valuable insights into these dynamics, but extrapolation from experiments to field conditions can only be done with caution.

Our growth performance data could also indicate a potential memory effect related to past frost events, i.e. enhanced frost tolerance due to frost‐induced epigenetic genome modification or transient physiological acclimation (Bruce et al., 2007; Chinnusamy et al., 2008; Goh et al., 2003). It is noteworthy that the combined negative effect of successive winter and spring frost events was of similar magnitude as the single effect size of the last spring frost event alone (Figure 4). This observation deviates from what would be expected in case of no acclimation (or even a carry‐over effect), where the negative effects of the different frost events would add up or even exceed the cumulative negative effect of repeated stress events, as suggested by previous studies (Kong & Henry, 2020; Walter et al., 2013). However, alternative explanations for our findings are also possible. One plausible causation is that the first frost event in spring selected more frost‐resistant individuals among the surviving plants, while acclimation played no role. Such a selection process could relate to genetic differences or varying phenotypic plasticity in relevant traits among the different individuals. In any case, our results align well with cell physiological studies in forb and grass species showing that cold‐acclimated plants reduce their metabolic activity less than non‐acclimated control plants (Knight et al., 1996) with the consequence that they are able to achieve a higher growth performance (e.g. Kong & Henry, 2020).

### 4.2 Winter frost‐induced delay in leaf‐out

The winter frost treatment delayed leaf‐out strongly in some of the experimental plants (Figure 6). The latest‐developing quarter of individuals enfolded their leaves more than 20 days after the corresponding fraction in the control group (Figure 6). In some individuals, sprouting was even delayed by up to 40 days (Figure 6). We can only speculate about the underlying physiological mechanisms that caused this extreme delay. One possible reason could be the need to build epicormic or adventitious buds after the putative loss of all active buds, but this hypothesis cannot be confirmed with our data. Before the application of the winter frost treatment in late February, the temperature did not drop below −7°C and no frost occurred during the last 8 days before the treatment, which is much warmer than during the frost treatment (Figure S2). Such a scenario of rapidly changing winter temperatures is quite realistic, as climate variability will increase and low daily minimum temperatures of −13°C at the end of February are not unlikely, despite global warming (Kodra et al., 2011; Francis et al., 2022; Figure S1). In this study, maximum frost tolerance may not have been fully achieved by the seedlings, as 2016/17 was a relatively warm winter in general, making the experimental frost (−13°C) a unique cold event in that winter. Moreover, the warmer period before the winter frost event may have triggered a de‐hardening process, likely reducing frost tolerance (Buchner & Neuner, 2011; Lenz et al., 2016; Vitra et al., 2017). Possible mechanism that delayed leaf‐out are frost‐induced xylem embolism which may lead to partial hydraulic failure (Beck et al., 2007; Lemoine et al., 1999), or frost damage to bud tissues, as leaf primordia are usually more frost‐sensitive than other plant organs including cambial meristems (Lenz et al., 2016). Embolism repair and the replacement of damaged leaf buds take time, which could well explain the delayed development. Studies measuring native xylem embolism and tissue damage through electrolyte leakage are needed to understand the factors that cause frost‐induced phenological delays.

Compared to the observed advancement of bud break by 1.3 days per 1°C in response to climate warming (Liu et al., 2022), a phenological delay of up to 40 days (Figure 6), caused by a one‐day frost event in late February, is unexpectedly large. Yet, despite this delay, nearly all plants flushed leaves eventually, even though some were so late that the annual photosynthetic carbon gain must have been greatly reduced. As a consequence, these individuals showed very little or no growth in the current year, as also demonstrated by Etzold et al. (2022).

Delayed leaf‐out may not only impair growth but also affect the plant's later performance in competition with other species. At the cold range limit of beech, it is likely that frost‐induced phenological delays weaken beech offspring in their interaction with more frost hardy seedlings of *Acer pseudoplatanus* L., *Betula pendula* Roth, *Prunus avium* L., and *Sorbus aucuparia* L. (Lenz et al., 2013; Luoranen et al., 2004), even though beech might be capable of frost acclimation. Competition experiments with tree species differing in frost hardiness are needed to examine the role of winter and spring frost for the fitness of tree offspring at the cold range limit of beech.

## 5 CONCLUSIONS

Our experimental study shows that beech seedlings increased their frost tolerance during subsequent frost episodes. Further, the high initial frost‐induced mortality suggests that winter and spring frosts were acting as effective environmental filters, so that more frost‐hardy individuals remained in the population. Nevertheless, the death toll from winter and spring frost events was severe with mortality rates reaching up to 75%, and the growth performance of the surviving individuals was negatively affected as well. The extremely late leaf emergence of a few individuals due to winter frost stress might well protect this seedling group from late spring‐frost events under field conditions, where such events are unlikely to occur so late in spring as in our experimental setting. However, these individuals with retarded phenology would be very weak competitors in the field due to their very low growth performance that they revealed in our study. This suggests that increased frost tolerance due to environmental filtering together with frost acclimation might not be sufficient to overcome the overall detrimental effects that are exerted by frost on the competitiveness of beech in cold habitats. Thus, the survival of beech seedlings and population regeneration may well be hampered by the limited frost sensitivity of the species' offspring, especially at the cold range limit.

## AUTHOR CONTRIBUTIONS

**Lena Muffler:** Conceptualization (equal); data curation (equal); formal analysis (supporting); investigation (equal); methodology (equal); project administration (lead); supervision (equal); validation (equal); visualization (equal); writing – original draft (lead); writing – review and editing (equal). **Robert Weigel:** Data curation (equal); formal analysis (lead); validation (equal); visualization (equal); writing – review and editing (equal). **Ilka Beil:** Investigation (equal); writing – review and editing (equal). **Christoph Leuschner:** Writing – review and editing (equal). **Jonas Schmeddes:** Investigation (equal); writing – review and editing (equal). **Juergen Kreyling:** Conceptualization (equal); formal analysis (supporting); funding acquisition (lead); investigation (equal); methodology (equal); project administration (supporting); resources (lead); supervision (equal); validation (equal); writing – review and editing (equal).

## CONFLICT OF INTEREST STATEMENT

All authors have no conflicts of interest to declare.

## Supporting information


Figures S1–S4

## Data Availability

The data can be found at the Göttingen Research Online repository at https://doi.org/10.25625/6IOSAT.
