# Supplementary material for: Winter and spring frost events delay leaf‐out, hamper growth and increase mortality in European beech seedlings, with weaker effects of subsequent frosts
Source: Ecol Evol. 2024 Jul 21;14(7):e70028. doi: 10.1002/ece3.70028 (PMC11260882; doi:10.1002/ece3.70028)
Supplement: Supplementary file 1 — Figures S1–S4 [file ECE3-14-e70028-s001.docx]

# Supporting Information

Muffler L, Weigel R, Beil I, Leuschner C, Schmeddes J, Kreyling J (2024) Winter and spring frost events delay leaf‐out, hamper growth and increase mortality in European beech seedlings, with weaker effects of subsequent frosts. Ecology and Evolution. [doi: 10.1002/ece370028](https://doi.org/10.1002/ece370028)


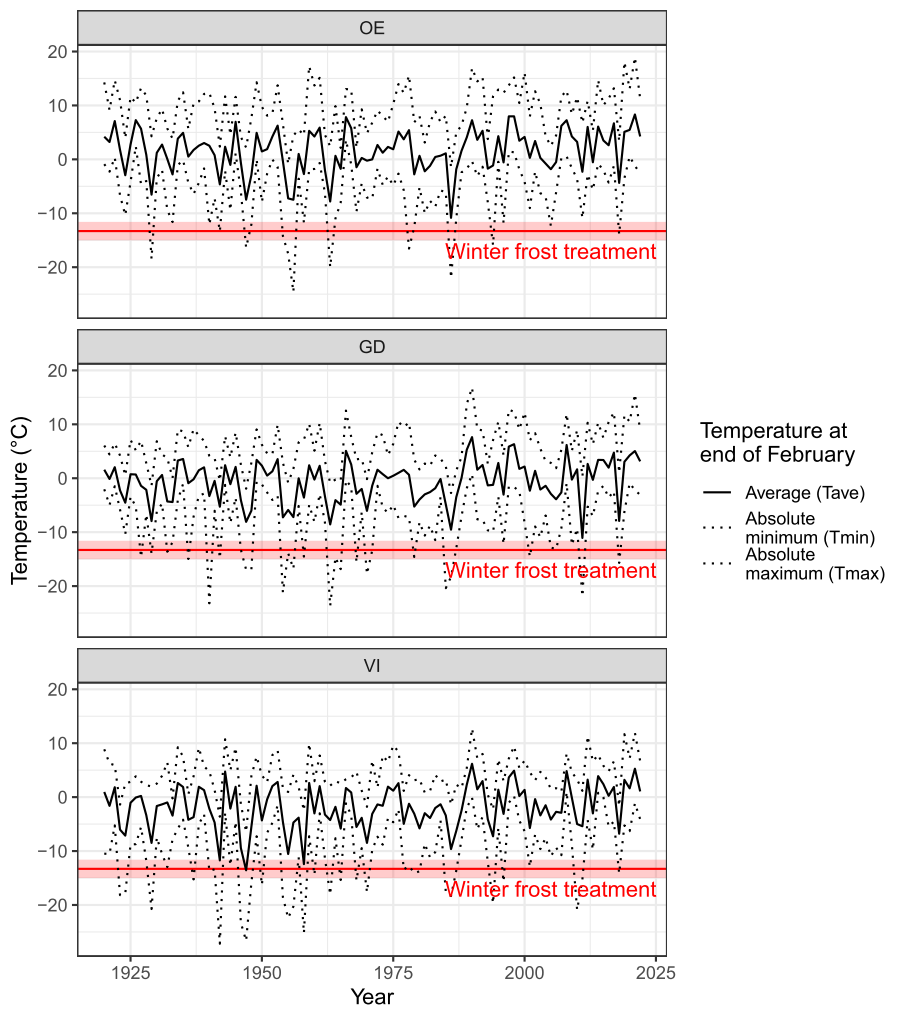


Figure S1 Averages, absolute minima, and absolute maxima of air temperatures in a static 9-day window at the end of February (February 20–28) in each year of the period 1920–2020 at the three locations where beech seeds were collected (Oerrel (OE), north-west Germany, Golub-Dobrzyn (GD), north-central Poland, and Visingsö (VI), southern Sweden). The temperature applied during the winter frost treatment is indicated by the red line.


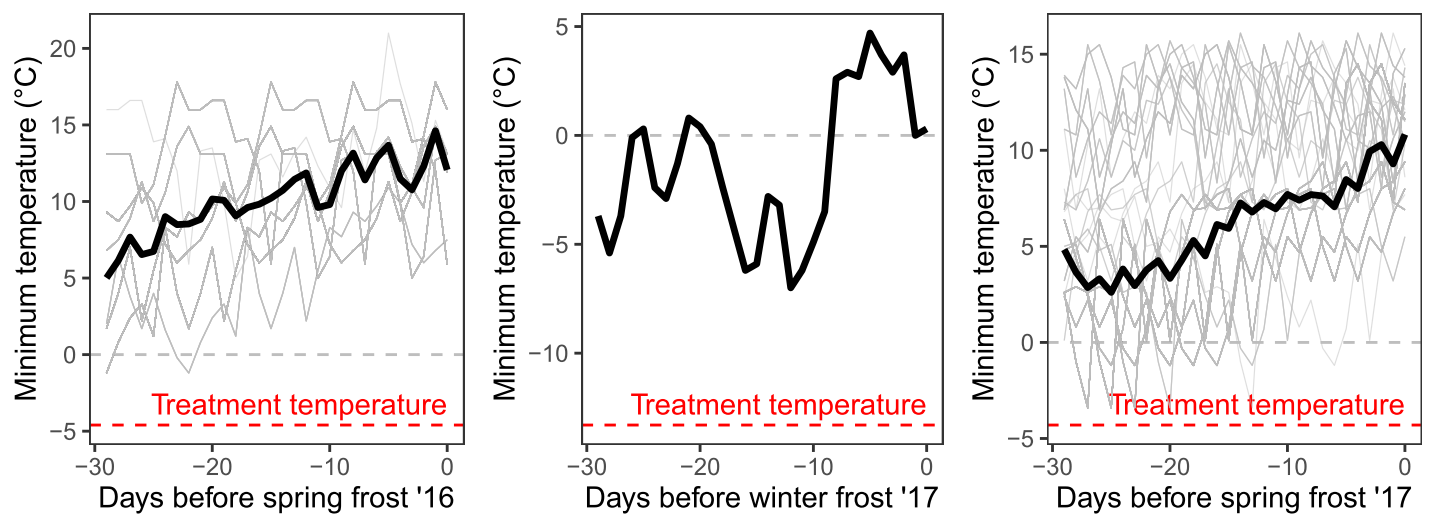


Figure S2 Ambient daily minimum air temperatures recorded 30 days before plants were transferred to the frost treatment (grey lines give temperature course of individual plants; black line is the average course) in relation to the frost treatment temperature during day “0” (red horizontal line). Temperature measured at 2 m above ground at the site of the common garden experiment in Greifswald with TRIX-8 thermistor–data logger units (LogTag, Lafayette, New Jersey, USA). Note that the temperature data were averaged over recordings at two points in opposing corners of the 50 m² experimental site in the garden with hourly resolution during the study (see continuous measurement in Figure S3). The graphs shown here represent this averaged time series based on the data shown in Figure S3 plotted for each individual and its individuals sampling date (day “0”). As the winter frost treatment was carried out at the same time for all individuals, only one temperature time series is shown here.


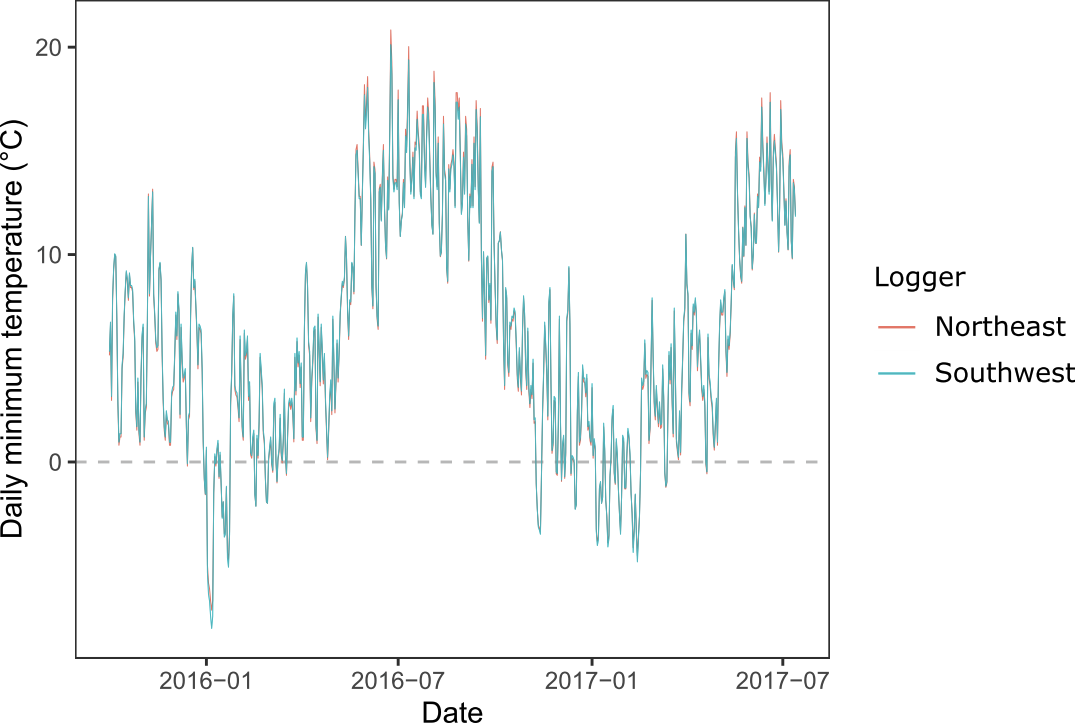
Figure S3 Ambient daily minimum air temperatures during autumn before the experiment until the date of last recorded leaf-out (1 October 2015 to 13 July 2017). Temperature measured in 2 m height above ground at two opposing corners of the common garden experiment in Greifswald with TRIX-8 thermistor–data logger units (LogTag, Lafayette, New Jersey, USA).


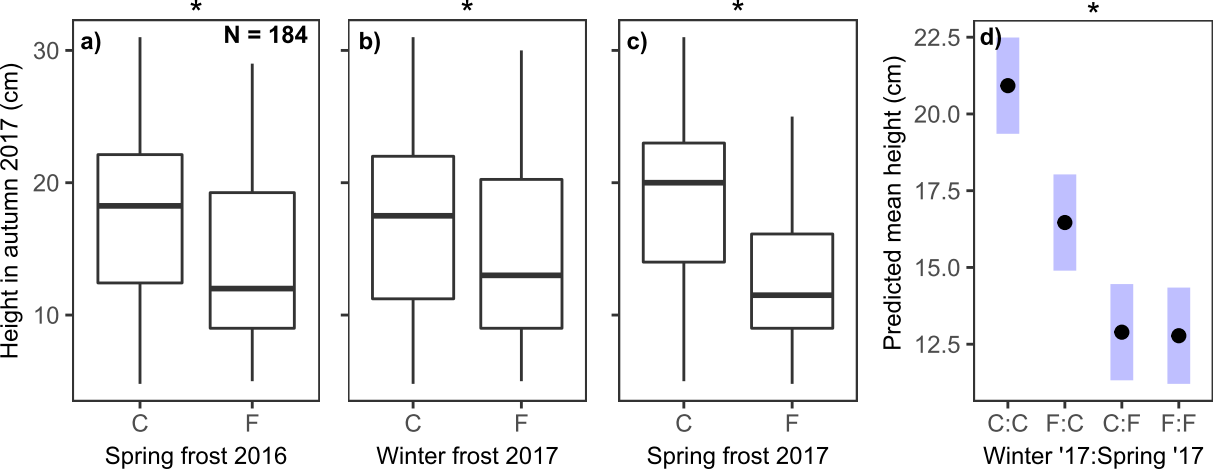


Figure S4 Plant height in autumn 2017 in dependence on the main treatment effects (C: control vs F: frost treatment) and d) Predicted mean height (cm) (with 95% confidence intervals) for the significant interaction of winter ’17 x spring ‘17 frost events. The main effects in a–c were isolated by ANOVA.
